# Gastric Perforation and Peritonitis Following Barium Swallow in a Patient With Prior Mechanical Ventilation and Stress Ulcer Prophylaxis: A Rare but Critical Complication

**DOI:** 10.1002/ccr3.70414

**Published:** 2025-04-07

**Authors:** Katrina J. Villegas, Islam Rajab, Hala Moussa, Mira Hallak, Patrick Michael

**Affiliations:** ^1^ Department of Internal Medicine St. Joseph's University Medical Center USA; ^2^ Department of Medicine An Najah National University Nablus Palestine

**Keywords:** abdominal imaging, barium swallow study, gastric ulcer perforation, GI prophylaxis, pneumoperitoneum, stress ulcer prophylaxis

## Abstract

Gastric perforation is a serious yet rare complication in critically ill patients on mechanical ventilation and GI prophylaxis. This report highlights an unexpected gastric perforation in a patient with a negative barium swallow study while receiving GI prophylaxis during intubation. A 65‐year‐old male with COPD and tobacco use disorder was treated with Oseltamivir for Influenza A infection, intubated for acute respiratory failure, and received IV pantoprazole for GI prophylaxis. After extubation and an unremarkable barium swallow study, he developed abdominal pain and tachycardia. Imaging showed free air under the diaphragm, and a CT scan indicated gastrointestinal perforation. Emergency surgery confirmed an anterior gastric ulcer perforation with purulent peritonitis. He was successfully treated with a washout and antibiotics and was discharged in stable condition. This case underscores the necessity of vigilance for abdominal symptoms and the potential for gastric perforation in critically ill patients despite GI prophylaxis, highlighting the importance of recognizing early signs of pneumoperitoneum.

**Summary**

- Gastric perforation remains a critical and potentially fatal complication in critically ill patients despite prophylactic measures against gastrointestinal distress.
- This case exemplifies the necessity for high vigilance in the assessment of subtle radiological signs such as free air under the diaphragm, which may initially be overlooked.
- Early and accurate recognition of these signs, coupled with prompt surgical intervention, is essential to manage this severe complication effectively and improve patient outcomes. Clinicians must maintain a high index of suspicion for gastrointestinal perforation in patients presenting with abdominal symptoms, even when preventive measures are in place.

## 1 Introduction

Gastric perforation is a rare but potentially life‐threatening complication in critically ill patients, particularly those who require mechanical ventilation. Stress ulcer prophylaxis, such as intravenous (IV) Pantoprazole, is commonly administered to prevent gastrointestinal bleeding in these patients. However, despite its widespread use, this intervention does not fully eliminate the risk of gastric perforation [1]. In this case report, we present a 65‐year‐old male with Influenza A infection treated with Oseltamivir and acute exacerbation of chronic obstructive pulmonary disease (COPD) who developed gastric perforation despite receiving appropriate gastrointestinal (GI) prophylaxis, a negative prior barium swallow study, and delayed recognition due to the subtle radiologic signs of pneumoperitoneum. The convergence of these factors—potential association of Oseltamivir and gastrointestinal manifestations, stress ulcer prophylaxis, recent barium swallow, and diagnostic challenges—underscores the complexity of managing critically ill patients and the need for heightened vigilance in identifying rare complications [2, 3].

## 2 Clinical History and Physical Examination

A 65‐year‐old male with a history of chronic obstructive pulmonary disease (COPD) and tobacco use disorder presented to the emergency department with symptoms of shortness of breath, productive cough, and yellow‐green sputum. He had a recent exposure to a sick contact and an increased use of Albuterol. Upon arrival, he exhibited hypertension, tachycardia, tachypnea, and was afebrile. His condition necessitated the initiation of bilevel positive airway pressure (BiPAP) for supportive management. The patient did not have any prior surgical abdominal history.

**FIGURE 1:**
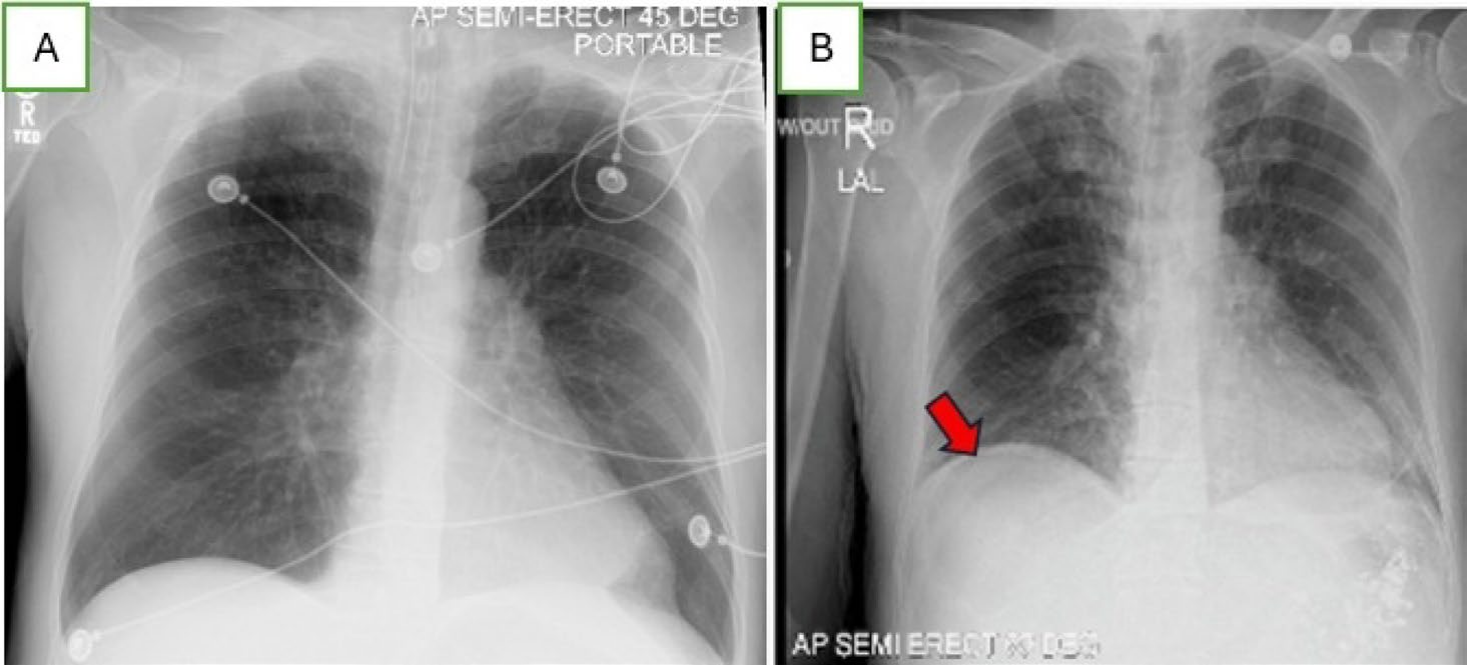
X‐ray of the chest and abdomen. (A) Anteroposterior chest radiograph obtained prior to extubation and before barium swallow evaluation. (B) Semi‐erect chest film showing free air under the right hemidiaphragm (red arrow).

Initial laboratory findings showed leukocytosis with a white blood cell count of 14.8 and a positive influenza A test. The chest X‐ray did not indicate infiltration, consolidation, or effusion (Figure 1A). The patient was treated with nebulized bronchodilators and intravenous Methylprednisolone. Despite these treatments, his hypercapnia progressed, leading to acute hypercapnic and hypoxic respiratory failure, which required intubation. In the Medical Intensive Care Unit (ICU), he received intravenous fluids, Oseltamivir, and broad‐spectrum antibiotics for suspected superimposed community‐acquired pneumonia, along with Pantoprazole for gastrointestinal prophylaxis during mechanical ventilation and steroid use. He was successfully extubated to BiPAP on Day 7 with stable hemodynamics, and gastrointestinal prophylaxis and steroids were discontinued. Pantoprazole was initiated for stress ulcer prophylaxis during the patient's critical illness. However, as the patient was extubated, no longer required steroid therapy, and was no longer considered critically ill, the decision was made to discontinue Pantoprazole.

## 3 Differential Diagnosis

After extubation, the patient failed a bedside swallow evaluation and underwent an unremarkable barium swallow study. Thereafter, the patient was tolerating oral intake. On Day 8, he experienced generalized abdominal pain and persistent tachycardia. A subsequent chest X‐ray performed on the same day as the onset of abdominal pain ruled out pulmonary infection but indicated subtle free air under the right hemidiaphragm (Figure 1B); yet this finding was not immediately recognized as an indication of gastrointestinal pathology, especially in the setting of recent intubation and mechanical ventilation.

**FIGURE 2:**
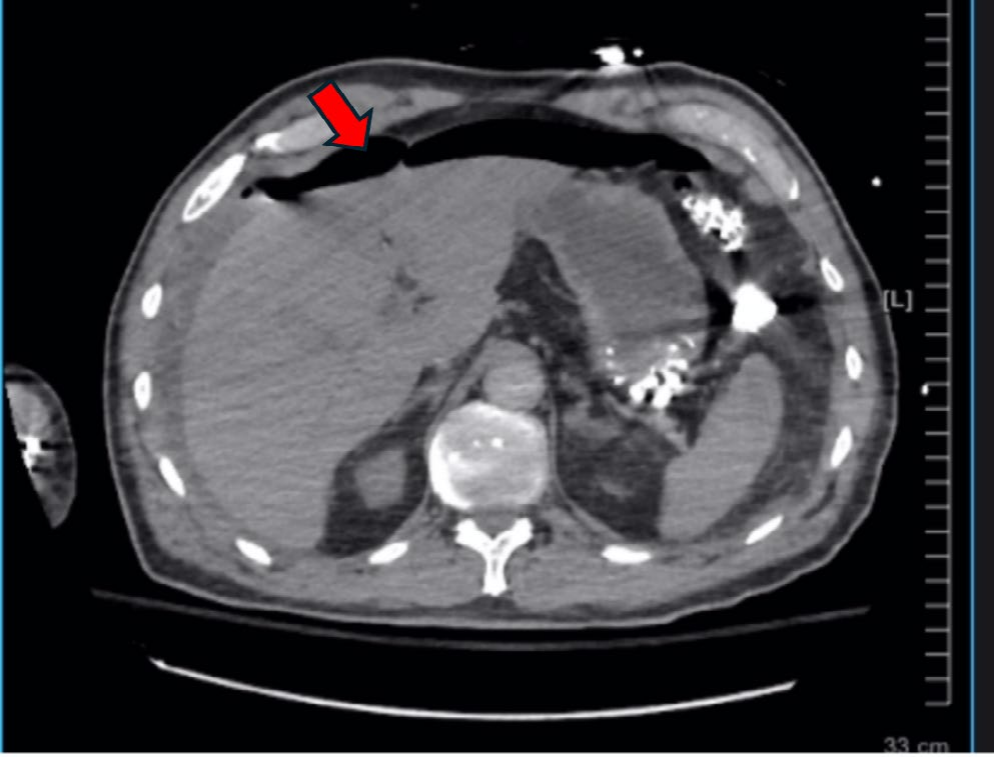
CT angiogram of the chest. CT scan shows extensive free air under the hemidiaphragm (red arrow). There is also free fluid in the abdomen.

During the continued assessment, given the sudden onset of hypoxia and tachycardia accompanied by an elevated D‐dimer, there was a high clinical suspicion of pulmonary embolism. A CT angiography of the chest, however, confirmed extensive free air under the diaphragm and ascites, leading to a diagnosis of gastrointestinal perforation (Figure 2). The radiology technician immediately flagged the presence of free air under the diaphragm to the bedside physician. Subsequently, the radiologist formally reviewed the images and communicated the findings of extensive pneumoperitoneum. This prompt communication facilitated urgent surgical intervention. The patient did not have a nasogastric tube or Dobhoff feeding catheter before the diagnosis of pneumoperitoneum and was not started on any enteral feeding before the emergency surgery.

## 4 Outcome and Follow‐Up

The patient underwent an exploratory laparotomy, with significant findings of purulent peritonitis and a 1.5 cm perforation on the anterior border of the stomach, located pre‐pylorically. Abdominal washout of the purulence was done, and a decision was made to proceed with a Graham patch repair of the gastric perforation. A nasogastric tube (NGT) was inserted intraoperatively. Postoperatively, he was managed in the Surgical ICU with antibiotics and supportive care. On postoperative Day 1, the NGT initially produced a small amount of reddish‐brown output, consistent with the expected findings. The patient's abdomen remained soft and minimally tender, with no signs of distension, rigidity, or guarding. The NGT was removed on postoperative Day 1, after confirming the absence of any complications and an adequate recovery trajectory. Oral feeding was initiated with sips of water, and the patient's tolerance was monitored closely. As the patient began to complain of hunger on postoperative Day 2 and demonstrated the ability to tolerate oral intake, feeding was gradually advanced. The patient continued to improve steadily, and by the time of discharge, he was able to tolerate a full diet. He was discharged in stable condition without any complications. His outpatient follow‐up was uneventful, with no complications reported.

## 5 Discussion

This case illustrates the unanticipated development of gastric perforation in a critically ill patient who was on mechanical ventilation and receiving GI prophylaxis with Pantoprazole. Gastric perforation is a rare but potentially fatal complication, and its occurrence despite preventive measures emphasizes the limitations of current strategies in critically ill patients [4]. The pneumoperitoneum was initially thought to be benign due to subtle findings on a chest X‐ray and recent intubation and mechanical ventilation. However, a later CT scan reviewed by a radiologist clearly showed extensive free air, confirming the diagnosis of perforation.

Initially, a chest X‐ray intended to check for pulmonary infection showed only subtle free air under the right hemidiaphragm (Figure 1B), not immediately recognized as indicative of perforation. This example underscores the diagnostic challenge of interpreting subtle signs on plain radiographs, which can delay the identification of critical conditions. It is well documented that patients with conditions such as COPD and those undergoing mechanical ventilation are at increased risk for stress‐related mucosal injury, leading to gastric ulcers and, less frequently, perforation [5]. These injuries result from reduced gastric perfusion, increased acid secretion, and mucosal barrier compromise in critically ill patients [6]. GI prophylaxis, while reducing the incidence of stress ulcers, does not entirely prevent the occurrence of such complications, particularly in the presence of additional risk factors such as steroid use or prolonged mechanical ventilation [7].

The gastric ulcer was classified as a type II ulcer according to the modified Johnson classification, which is associated with acid hypersecretion [8]. While gastric perforation cases have been reported in the literature, what sets this case apart is the negative barium swallow study just a day before the perforation and the delayed recognition of the subtle pneumoperitoneum on imaging.

To our knowledge, there is no reported association between Oseltamivir and gastric perforation, although a study reported a rare case of a pediatric patient who developed a perforated peptic ulcer following influenza A infection, which could have been an adverse event related to Oseltamivir administration [9]. In this case, the patient had been treated with Oseltamivir for influenza A infection. While oseltamivir is generally well‐tolerated, it has been associated with transient gastrointestinal symptoms [10]. The combination of a recent Oseltamivir use and a negative prior barium study in a patient subsequently diagnosed with a type II gastric ulcer presents a unique clinical scenario, although further research is needed to explore the potential association between Oseltamivir and gastrointestinal complications.

The case emphasizes the importance of timely communication between radiology and clinical teams in emergency situations. After the CT scan, the radiology technician alerted the bedside physician about free air under the diaphragm, but the formal report from the radiologist followed shortly thereafter. This scenario highlights the need for clinicians to quickly respond to preliminary imaging findings to ensure patient safety.

Additionally, the diagnostic decision‐making in this case was influenced by the concern of pulmonary embolism (PE) over a gastrointestinal pathology. The patient's sudden onset of hypoxia, tachycardia, and elevated D‐dimer raised strong concern for PE. The clinical presentation, including the patient's history of COPD and the recent critical illness, prompted an initial diagnostic focus on PE. Despite the patient's complaint of abdominal pain, the physical examination did not suggest a surgical abdomen or peritonitis, and there were no overt signs of gastrointestinal distress. This led to a lower immediate concern for a gastrointestinal pathology, although the possibility remained. The elevated D‐dimer, while not specific, further supported the suspicion of PE. Given the clinical presentation, a CT angiography of the chest was performed to evaluate for PE, but instead revealed extensive pneumoperitoneum, ultimately guiding the diagnosis toward gastrointestinal perforation.

Our case highlights that even with appropriate prophylactic therapy, early detection of gastric perforation remains challenging, particularly in patients who do not present with overt abdominal symptoms. The subtle appearance of pneumoperitoneum in early imaging can delay the diagnosis, resulting in the need for emergent surgical intervention. Subtle free air under the diaphragm is often difficult to appreciate on chest X‐rays, emphasizing the need for a high index of suspicion and early utilization of advanced imaging modalities such as CT, which are more sensitive in identifying small amounts of extraluminal air. A broader understanding of the limitations of diagnostic studies, including barium swallow, is also crucial, as these studies may miss small perforations or other critical findings [11].

The timing of clinical deterioration and delayed surgical intervention is consistent with the pathophysiology of gastric perforation, which can rapidly progress to purulent peritonitis if not addressed promptly [12]. Prompt surgical exploration is essential to confirm the diagnosis and perform necessary interventions [12]. In this case, the absence of barium leakage into the abdominal cavity, despite the prior barium swallow study, reinforces the need to carefully interpret imaging findings and understand that no single diagnostic test is definitive in such complex scenarios. Implementing standardized monitoring protocols, including periodic abdominal imaging and scoring systems for critically ill patients, may help detect complications earlier.

This case underscores the need for heightened awareness and vigilance in critically ill patients, even in the absence of clear diagnostic findings. Multidisciplinary collaboration between radiologists and surgeons is essential in ensuring early recognition and intervention. Clinicians should consider gastrointestinal perforation in the differential diagnosis for any patient who develops abdominal symptoms, particularly when subtle radiologic signs such as free air are noted on imaging studies [13]. Early recognition and management of this potentially fatal complication can significantly impact patient outcomes. Finally, this case highlights the need for future research into optimizing GI prophylaxis and refining diagnostic approaches to minimize the risk of such complications in critically ill populations.

This case highlights the rare but potentially fatal complication of gastric perforation in a critically ill patient despite GI prophylaxis with Pantoprazole and a negative barium swallow study. It emphasizes the need to consider all contributing factors, including recent antiviral therapy, and the importance of early recognition of subtle imaging signs, such as pneumoperitoneum, to avoid delayed diagnosis. Clinicians must remain vigilant for abdominal complications, even without clear symptoms, and prioritize timely intervention to prevent further morbidity. Multidisciplinary collaboration and careful imaging interpretation are crucial in managing such cases.

## Author Contributions

**Katrina J. Villegas:** conceptualization, supervision, visualization, writing – original draft, writing – review and editing. **Islam Rajab:** conceptualization, supervision, visualization, writing – original draft, writing – review and editing. **Hala Moussa:** visualization, writing – original draft. **Mira Hallak:** data curation, writing – original draft, writing – review and editing. **Patrick Michael:** visualization, writing – original draft.

## Ethics Statement

Our institution does not require ethical approval for reporting individual cases. This study was performed in accordance with the Helsinki Declaration of 1964 and its later amendments.

## Consent

Written informed consent was obtained from the patient for their anonymized information to publish this report in accordance with the journal's patient consent policy.

## Conflicts of Interest

The authors declare no conflicts of interest.

## Data Availability

No new data are available. Information and data that support the findings of this study are derived from previously published sources, which have been cited appropriately within the text of the manuscript.
